# Efficacy and safety of high-power short-duration ablation for atrial fibrillation: a systematic review and meta-analysis of randomized controlled trials

**DOI:** 10.1007/s10840-024-01782-2

**Published:** 2024-03-09

**Authors:** Ahmed Mazen Amin, Ramy Ghaly, Ahmed A. Ibrahim, Mohamed Ahmed Ali, Omar Almaadawy, Amr Elzahaby, Mohamed Abuelazm, Basel Abdelazeem, Muhammad Bilal Munir

**Affiliations:** 1 Faculty of Medicine, Mansoura University, Mansoura, Egypt; 2 Internal Medicine, University of Missouri-Kansas City, Kansas City, MO USA; 3 Faculty of Medicine, Menoufia University, Menoufia, Egypt; 4 Qena Faculty of Medicine, South Valley University, Qena, Egypt; 5 Internal Medicine, MedStar Health, Baltimore, MD USA; 6 Faculty of Medicine, Tanta University, Tanta, Egypt; 7 Department of Cardiology, West Virginia University, Morgantown, WV USA; 8 Section of Electrophysiology, Division of Cardiology, Department of Medicine, University of California Davis, Sacramento, CA USA

**Keywords:** Atrial fibrillation, Ablation, Pulmonary vein isolation, HPSD, High Power

## Abstract

**Background:**

High-power short-duration (HPSD) ablation has emerged as an alternative to conventional standard-power long-duration (SPLD) ablation. We aim to assess the efficacy and safety of HPSD versus SPLD for atrial fibrillation (AF) ablation.

**Methods:**

A systematic review and meta-analysis of randomized controlled trials (RCTs) retrieved from PubMed, WOS, SCOPUS, EMBASE, and CENTRAL were performed through August 2023. We used RevMan V. 5.4 to pool dichotomous data using risk ratio (RR) and continuous data using mean difference (MD) with a 95% confidence interval (CI). PROSPERO ID: CRD42023471797.

**Results:**

We included six RCTs with a total of 694 patients. HPSD was significantly associated with a decreased total procedure time (MD: -22.88 with 95% CI [-36.13, -9.63], P = 0.0007), pulmonary vein isolation (PVI) time (MD: -19.73 with 95% CI [-23.93, -15.53], P < 0.00001), radiofrequency time (MD: -10.53 with 95% CI [-12.87, -8.19], P < 0.00001). However, there was no significant difference between HPSD and SPLD ablation with respect to the fluoroscopy time (MD: -0.69 with 95% CI [-2.00, 0.62], P = 0.30), the incidence of esophageal lesions (RR: 1.15 with 95% CI [0.43, 3.07], P = 0.77), and the incidence of first pass isolation (RR: 0.98 with 95% CI [0.88, 1.08], P = 0.65).

**Conclusion:**

HPSD ablation was significantly associated with decreased total procedure time, PVI time, and radiofrequency time compared with SPLD ablation. On the contrary, SPLD ablation was significantly associated with low maximum temperature.

**Graphical Abstract:**

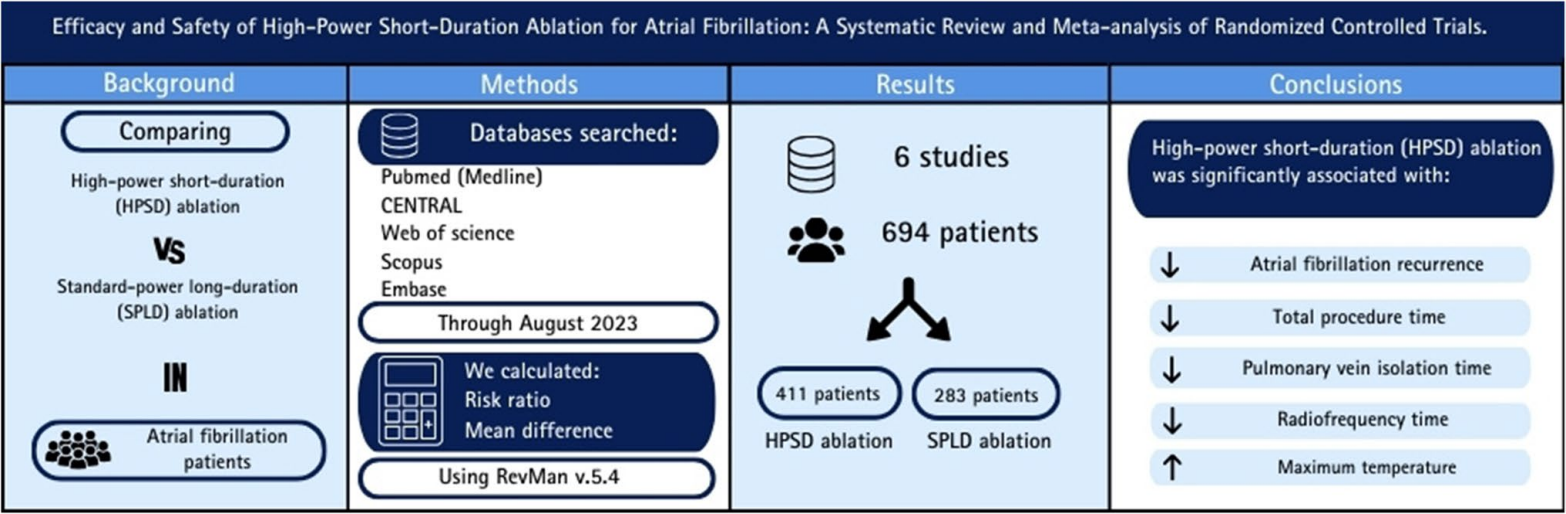

**Supplementary Information:**

The online version contains supplementary material available at 10.1007/s10840-024-01782-2.

## Introduction

Current guidelines recommend catheter ablation for patients with symptomatic atrial fibrillation (AF) who are refractory or intolerant to anti-arrhythmic drugs (AAD). Additionally, catheter ablation may serve as an initial rhythm-control strategy for certain patients experiencing symptomatic paroxysmal AF, with the goal of alleviating symptoms and mitigating progression to persistent AF [1]. Catheter ablation was shown to be more effective in maintaining normal sinus rhythm than AAD [2–7].

The prime triggers behind AF initiation and perpetuation are ectopic electrical discharges stemming from pulmonary veins in the left atrium [8]. Therefore, ablation procedures aim to isolate the pulmonary vein by creating circumferential ablation lesions that encircle the pulmonary veins ostia. Radiofrequency ablation (RFA) and cryo-ablation are the commonly used and approved ablation techniques with comparable efficacy [9, 10].

RFA delivers thermal energy to cauterize cardiac tissue. The efficacy and safety of RFA depend on achieving full-thickness and durable lesions without causing collateral damage, such as esophageal thermal injury (ETI) and pericardial effusion due to cardiac perforation. The key factors influencing the design of the created lesion, including its size and depth, are power, duration, catheter stability, and contact force [11]. Recently, high-power short-duration (HPSD) ablation (40–50 W) has emerged as an alternative to conventional standard-power long-duration (SPLD) ablation (25–35 W). Retrospective studies comparing HPSD to SPLD ablation have suggested shorter procedure times with comparable efficacy and safety profiles with HPSD lesions [12–14]. Theoretically, the HPSD technique delivers more significant resistive heating to the surrounding myocardium, whereas the SPLD technique delivers more significant conductive heating within the distal myocardium and surrounding structures [15]. Several randomized controlled trials (RCTs) have been conducted to compare HPSD and SPLD RFA outcomes [16–21].

To thoroughly assess the existing data and aid in clinical decision-making, we conducted this systematic review and meta-analysis to investigate outcomes, such as procedure duration, recurrence rates, first-pass isolation rates, and safety profile between HPSD and SPLD in AF patients undergoing RFA.

## Methodology

### Protocol registration

This study complied with the PROSPERO protocol, registered under ID: CRD42023471797. We adhered to the PRISMA statement guidelines for systematic reviews and meta-analysis [22] and the Cochrane Handbook for Systematic Reviews and Meta-Analysis [23] guidelines.

### Data sources & search strategy

Data Sources & Search Strategy: PubMed (Medline), EMBASE, Web of Science, SCOPUS, and Cochrane Central Register of Controlled Trials (CENTRAL) were scoured by two researchers (A.M.A. and M.A.) from their inception to August 2023. A distinct search approach was applied to each database, as detailed in (Table S1).

### Eligibility criteria

RCTs followed the following Population, Intervention, Comparison, and Outcomes (PICO) criteria were included: population (P): patients with paroxysmal and persistent AF; intervention (I): HPSD; control (C): SPLD; outcome (O): our primary outcomes were total procedure time, pulmonary vein isolation (PVI) time, radiofrequency (RF) application time, fluoroscopy time, and esophageal lesions while secondary outcomes included: AF recurrence, atrial flutter (AFL)/ atrial tachycardia (AT) recurrence, atrial arrhythmias recurrence, first pass left pulmonary vein (LPV) isolation, first pass right pulmonary vein (RPV) isolation, and first pass isolation. In addition, safety outcomes included any complications and maximum temperature. Studies were excluded if they were: (1) letters, theses, editorials, book chapters, cohort studies, case series, case reports, single-arm studies, animal studies, in vitro research, or preliminary studies; (2) research where data was solely sourced from abstracts.

### Study selection

The initial step was to screen titles and abstracts independently by three researchers (O.A., M.A.A., and A.A.) using the Covidence online platform. Post deduplication removal, dual independent screening was applied to each citation. The same reviewers undertook the full-text review, with discrepancies settled by a third reviewer (A.M.A. and M.A.) in accordance with our previous eligibility criteria.

### Data extraction

A standardized Excel extraction template, which had undergone preliminary testing, was employed by four reviewers (O.A., M.A., and A.A.) to retrieve pertinent information from the selected studies. This encompassed: (1) a summary section detailing the study's design, nation of origin, the number of participating centers, total participants, aims for the intervention and control, techniques employed for both, power specifications, essential inclusion prerequisites, primary results, and the span of the follow-up; (2) baseline information (Number of patients in each group, sex (male), age (Years), BMI, CHA_2_DS_2_-VASc score, left ventricular ejection fraction (LVEF), AF type (paroxysmal or persistent). We also included comorbidities, which include hypertension, diabetes mellitus, ischemic heart disease (IHD), or coronary artery disease, obstructive sleep apnea, and stroke; and (3) study outcomes (AF Recurrence, AFL/AT recurrence, atrial arrhythmias recurrence, first pass LPV isolation, first pass RPV isolation, and first pass isolation, total procedure time, PVI time, RF application time, fluoroscopy time, maximum temperature. We also looked at safety data, which included any complications and esophageal lesions. Conflicts were discussed and resolved by consensus.

### Risk of bias and certainty of evidence

Three reviewers (M.A., O.A., and A.A.) independently used the Cochrane ROB2 tool [24] for quality assessment. The domains that were evaluated included the risk of bias resulting from the randomization process, the risk of bias due to deviation from the intended intervention, the risk of bias due to missing outcome data, the risk of bias in measuring the outcome, and the risk of bias in selecting the reported results. The reviewers resolved any conflicts by consensus.

M.A. used the Grading of Recommendations Assessment, Development, and Evaluation (GRADE) guidelines [25, 26] to evaluate the certainty of evidence for each outcome. The decisions made were justified and recorded.

### Statistical analysis

RevMan v5.3 was used to run the statistical analysis [27]. To pool the results of dichotomous outcomes, we used the risk ratio (RR), while for the continuous outcomes, we used the mean difference (MD), both with a 95% confidence interval (CI). We performed both the Chi-square and I-square tests to evaluate heterogeneity, where the Chi-square test detects the presence of heterogeneity, and the I-square test evaluates its degree. I-square was interpreted In accordance with the Cochrane Handbook (chapter nine) [23] as follows: heterogeneity is not significant for 0–40 percent, moderate for 30–60 percent, substantial for 50–90 percent, and considerable for 75–100 percent. We considered an alpha level below 0.1 for the Chi-square test to detect significant heterogeneity. A leave-one-out sensitivity analysis was employed to resolve the heterogeneity by excluding each study one time from the pooled analyzed studies.

We made a subgroup analysis between studies that used ≥ 50 W versus < 50 W in the HPSD arm.

## Results

### Search results and study selection

**Fig. 1:**
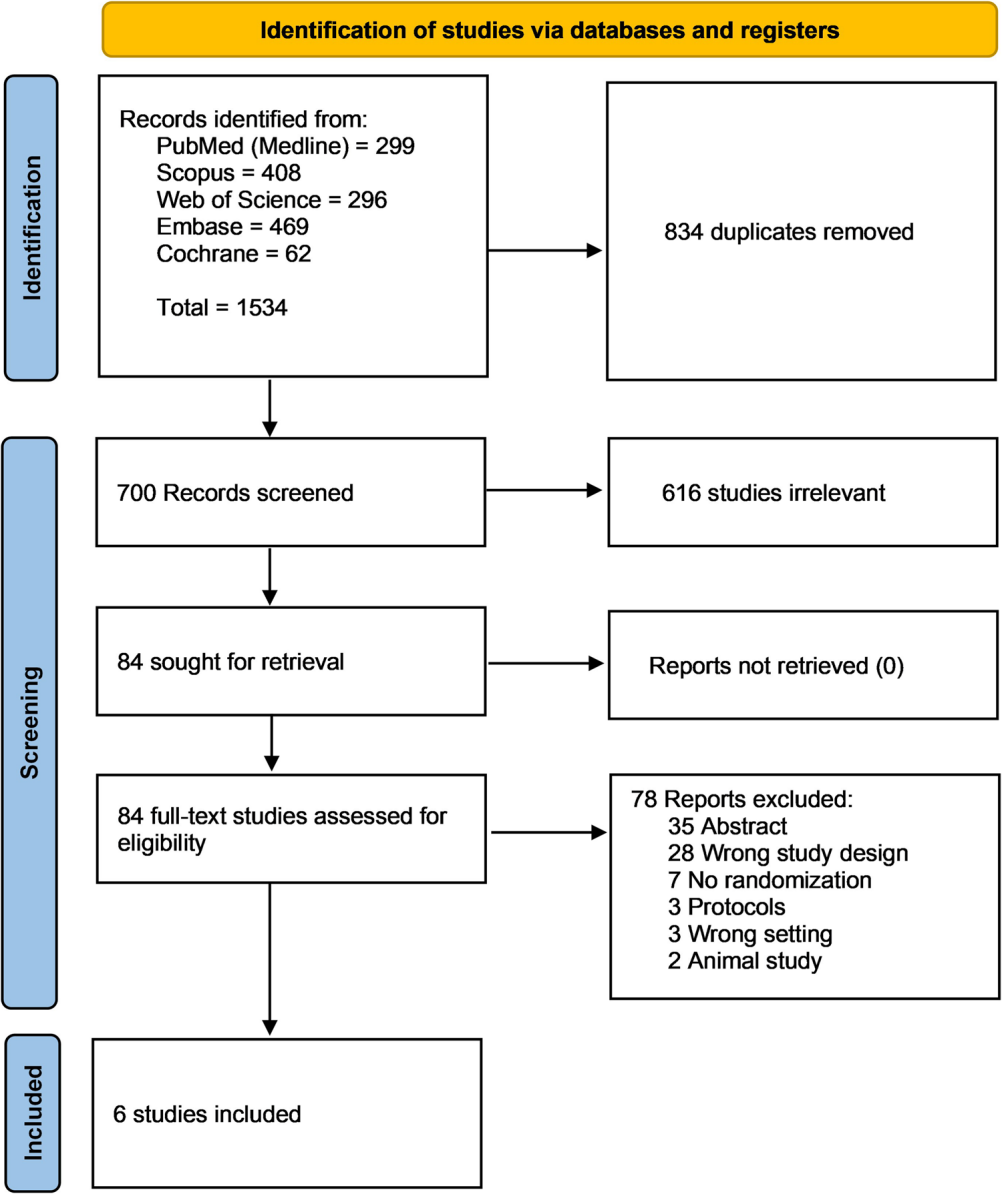
PRISMA flow chart of the screening process

Using our search strategy, we searched (PubMed, Cochrane, Embase, Web of Science, and Scopus), and reached 1534 studies. A total of 834 duplicate studies were removed, and 616 were excluded after screening their titles and abstracts. We reviewed the full text of the remaining 84 studies; 78 were removed from the final assessment and subsequent data analysis (Fig. 1).

### Characteristics of included studies

In brief, six RCTs [16–21] were included for the final review and data analysis. The total number of patients was 694, with 411 patients in the HPSD group and 283 in the SPLD group. More details about the trials’ inclusion criteria, ablation guidance, and ablation target with baseline trials’ participants' comorbidities are outlined in (Table 1, 2, and S2-S3).

**Table 1:** Summary characteristics of the included RCTs

Study ID	Study Design	Country	Total Participants	Intervention power used	Control power used	Additional ablations	Type of AF included	Ablation procedure cut-off		First or repeated ablation	Primary Outcome	Follow-up duration
								HPSD	SPLD			
Chieng et al. 2022 (Hi-Lo HEAT trial) [16]	Multi-center, double-blinded, RCT	Australia	88	40 W	25 W	Posterior wall isolation in persistent AF and some patients underwent CTI ablation	Paroxysmal and persistent (AF lasting ≥ 7 days) AF	Ablation was terminated when either of the following occurred:(i) AI of 400 or LSI of 4 was achieved; or (ii) luminal oesophageal temperature exceeded ≥ 38 °C, or there was a steep rise of > 1 °C within 5 s	Ablation was terminated when either of the following occurred:(i) AI of 400 or LSI of 4 was achieved; or (ii) luminal oesophageal temperature exceeded ≥ 38 °C, or there was a steep rise of > 1 °C within 5 s	First time ablation	The incidence of ETI in the HPSD and LPLD groups	12 months
Francke et al. 2021 [17]	Single‐center, single-blinded, RCT	Germany	120	50 W	20 W at the posterior and inferior wall, and with 40 W at the roof and anterior wall	Some patients underwent additional ablations such as CTI ablation	Paroxysmal and persistent AF	AI targets of 400 and 550 were used for posterior and anterior lesions, respectively	AI targets of 400 and 550 were used for posterior and anterior lesions, respectively	First time ablation	The differences in total procedural time, the total RF application time, and the time to PVI using the AI‐guided fixed HPSD protocol compared to the standard CLOSE protocol	12 weeks
Lee et al. 2023 (SHORT-AF) [18]	Dual center, single-blinded, RCT	USA	60	50 W	25–30 W	No patients underwent additional ablation beyond PVI	Paroxysmal and persistent (< 1 year) AF	For CARTO, a Surpoint ablation index of 450 to 550 on the anterior, LA appendage ridge, or septal aspects and 350 to 400 on the posterior aspects of the PV antra was targeted. For Ensite, a lesion size index of 5.5 to 6.0 on the anterior, LA appendage ridge, or septal aspect and 4.5 to 5.0 on the posterior aspects of the PV antra were targeted	For CARTO, a Surpoint ablation index of 450 to 550 on the anterior, LA appendage ridge, or septal aspects and 350 to 400 on the posterior aspects of the PV antra was targeted. For Ensite, a lesion size index of 5.5 to 6.0 on the anterior, LA appendage ridge, or septal aspect and 4.5 to 5.0 on the posterior aspects of the PV antra were targeted	First time ablation	time to achieve PVI	12 months
O’Neill et al. 2023 (POWER PLUS) [19]	Multicenter, open-label, RCT	Belgium, Switzerland, Austria and Netherlands	180	90 W	35/50W	Some patients underwent CTI ablation	Paroxysmal and persistent AF	PVI was performed at a power of 90 W over 4 s	cutoff temperature 50 °C), AI values were targeted to ≥ 550 at the anterior wall and ≥ 400 posteriorly, and at the roof and inferior aspect of the veins with an inter-tag distance of ≤ 6 mm. In the case of an intraesophageal temperature rise > 38.5 °C during posterior wall ablation, RF delivery was discontinued at an AI value of 300 and a cooling time was respected	First time ablation	procedure time	6 months
Shin et al. 2021 [20]	Multicenter, single-blinded RCT	Korea	150	40 W—50 W	30 W	Additional ablation including the box lesion and/or lateral peri-mitral line was performed in a total of 31 patients	paroxysmal and non- paroxysmal AF	In all patients, we applied 25–30W without exceeding CF of 20 g for a maximum of 20 s to the posterior segments of PV antra and posterior-inferior line between each lower PV	In all patients, we applied 25–30W without exceeding CF of 20 g for a maximum of 20 s to the posterior segments of PV antra and posterior-inferior line between each lower PV	First time ablation	procedure and ablation time	12 months
Wielandts et al. 2022 (POWER-AF) [21]	Single-center RCT	Belgium	100	45 W	35 W	Some patients underwent CTI ablation	Paroxysmal AF	Radiofrequency was delivered until an AI ≥ 550 at the anterior wall and ≥ 400 everywhere else and with a CF ≤ 30 g	Radiofrequency was delivered until an AI ≥ 550 at the anterior wall and ≥ 400 everywhere else and with a CF ≤ 30 g	first CLOSE-guided pulmonary vein isolation	application time and procedure time,	6 months

*ETI* esophageal thermal injury, *HPSD* high-power short-duration, *LPLD* low-power long-duration, *RF* radio-frequency, *PVI* pulmonary vein isolation, *AI* ablation index, *CTI* Cavotricuspid isthmus ablation, *LA* Left atrium, *NA* not available

**Table 2:** Baseline characteristics of the participants

Study ID	Number of patients in each group		Age (Years), Mean (SD)		Gender (Male), N. (%)		BMI, Mean (SD)		CHA2DS2VAS, Mean (SD)		LVEF, Mean (SD)		AF type N. (%)			
	Intervention	Control	Intervention	Control	Intervention	Control	Intervention	Control	Intervention	Control	Intervention	Control	Paroxysmal		Persistent	
													Intervention	Control	Intervention	Control
Chieng et al. 2022 (Hi-Lo HEAT trial) [16]	44	44	62.9 (8.2)	59.7 (10.0)	31 (70.5)	30 (68.2)	29.2 (5.6)	29.2 (4.8)	2 (2)	1.5 (1.5)	55.4 (13.0)	54.6 (11.4)	15 (34.1)	21 (47.7)	29 (65.9)	23 (52.3)
Francke et al. 2021 [17]	100	20	66.4 (10)	66.4 (10)	60 (60)	7 (35)	NA	NA	2.8 (1.5)	3.2 (1.5)	54.2 (13.3)	59.5 (5.5)	49 (49)	9 (45)	51 (51)	11 (55)
Lee et al. 2023 (SHORT-AF) [18]	29	31	67.3 (8.6)	63.3 (7)	20 (69)	25 (81)	29.8 (6.8)	28.6 (4.4)	2 (1.6)	2 (1.6)	58.3 (11.7)	58.3 (3.9)	17 (59)	17 (55)	12 (41)	14 (45)
O’Neill et al. 2023 (POWER PLUS) [19]	90	90	64.2 (8.9)	62.3 (10.8)	61 (67.8)	59 (65.6)	26.6 (3.1)	26.9 (4.3)	1.7 (2.3)	1.5 (0.8)	82 (91.1)	83 (92.2)	64 (71.1)	75 (83.3)	26 (28.9)	15 (16.7)
Shin et al. 2021 [20]	100	50	57.9 (9.4)	58.7 (11.1)	81 (81)	33 (66)	24.4 (2.85)	24.6 (2.7)	1.65 (1.4)	1.7 (1.6)	55.65 (11.7)	58.9 (8.3)	48 (48)	24 (48)	52 (52)	26 (52)
Wielandts et al. 2022 (POWER-AF) [21]	48	48	64 (11)	61 (11)	32 (66.7)	33 (69)	26.4 (4.2)	26.8 (4)	1.3 (2.3)	1.3 (2.3)	NA	NA	48 (100)	48 (100)	0 (0)	0 (0)

*N.* number; *SD* standard deviation; *BMI* body mass index; *LVEF* left ventricular ejection fraction; *AF* atrial fibrillation

### Risk of bias and certainty of evidence

**Fig. 2:**
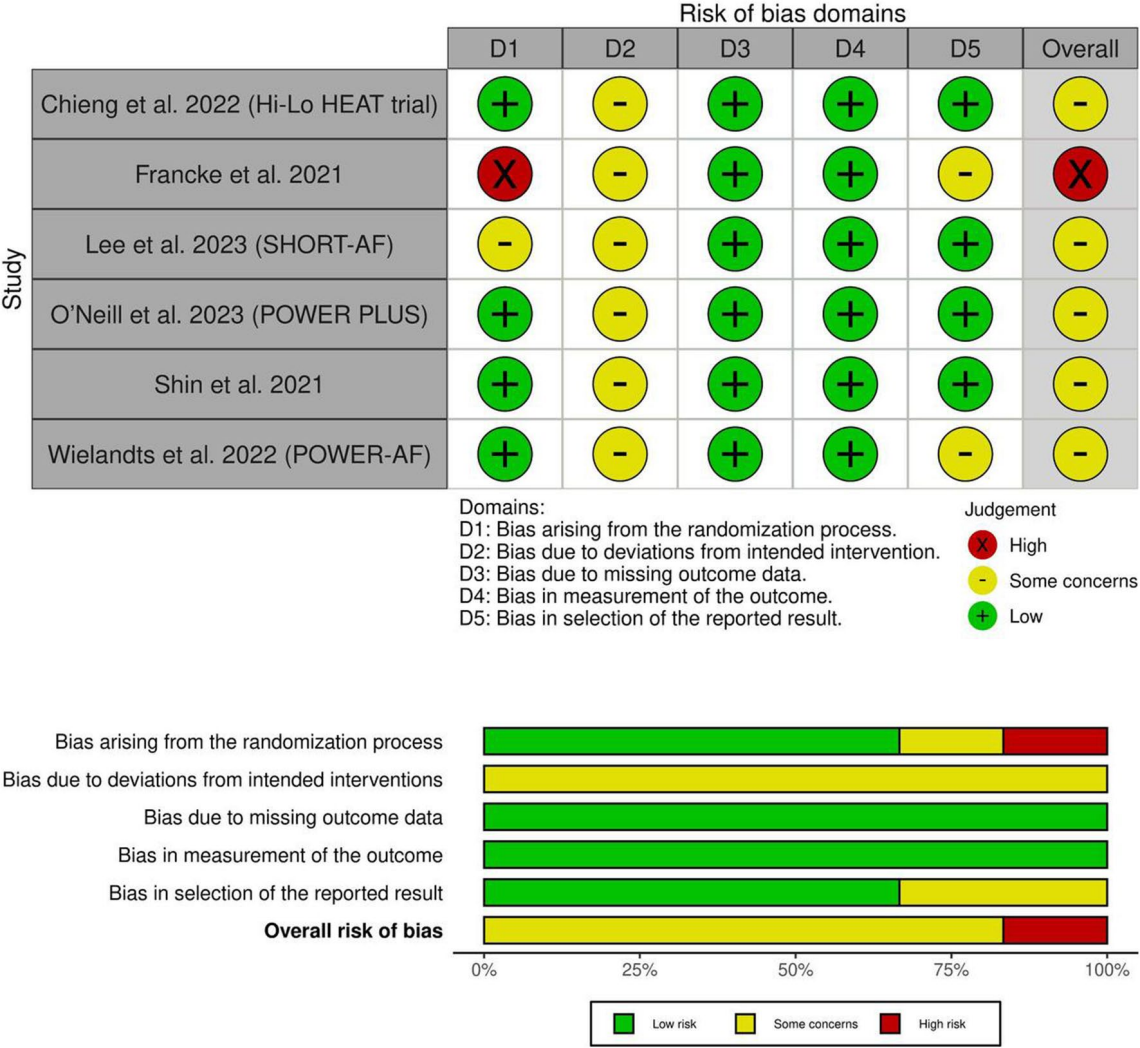
Quality assessment of risk of bias in the included trials. The upper panel presents a schematic representation of risks (low = green, unclear = yellow, and high = red) for specific types of biases of each study in the review. The lower panel presents risks (low = green, unclear = yellow, and high = red) for the subtypes of biases of the combination of studies included in this review

We used Cochrane RoB 2 to assess the risk of bias. One study had an overall high risk of bias [17], while five studies had an overall some concerns [16, 18–21]. Results are shown in (Fig. 2). In addition, the authors’ descriptions of the consequences of their decisions are outlined in (Table S4). Finally, the certainty of evidence is demonstrated in a GRADE evidence profile (Table 3).

**Table 3:** GRADE evidence profile

Certainty assessment							Summary of findings				
Participants (studies) Follow-up	Risk of bias	Inconsistency	Indirectness	Imprecision	Publication bias	Overall certainty of evidence	Study event rates (%)		Relative effect (95% CI)	Anticipated absolute effects	
							With LPLP Ablation	With HPSD Ablation		Risk with LPLP Ablation	Risk difference with HPSD Ablation
**AF Recurrence**											
450 (4 RCTs)	serious^a^	not serious	not serious	very serious^b^	none	⨁◯◯◯ Very low	29/160 (18.1%)	28/290 (9.7%)	**RR 0.60** (0.37 to 0.98)	181 per 1,000	**73 fewer per 1,000** (from 114 to 4 fewer)
**Recurrence of atrial tachycardia/atrial flutter**											
234 (2 RCTs)	serious^a^	not serious	not serious	extremely serious^b^	none	⨁◯◯◯ Very low	9/92 (9.8%)	8/142 (5.6%)	**RR 0.61** (0.24 to 1.54)	98 per 1,000	**38 fewer per 1,000** (from 74 fewer to 53 more)
**Recurrence of atrial arrhythmias**											
474 (4 RCTs)	serious^a^	not serious	not serious	very serious^b^	none	⨁◯◯◯ Very low	49/213 (23.0%)	52/261 (19.9%)	**RR 0.91** (0.64 to 1.28)	230 per 1,000	**21 fewer per 1,000** (from 83 fewer to 64 more)
**First pass LPV**											
484 (4 RCTs)	serious^a^	not serious	not serious	not serious	none	⨁⨁⨁◯ Moderate	181/202 (89.6%)	255/282 (90.4%)	**RR 1.00** (0.93 to 1.07)	896 per 1,000	**0 fewer per 1,000** (from 63 fewer to 63 more)
**First pass RPV**											
484 (4 RCTs)	serious^b^	serious^c^	not serious	serious^b^	none	⨁◯◯◯ Very low	163/202 (80.7%)	234/282 (83.0%)	**RR 1.06** (0.88 to 1.27)	807 per 1,000	**48 more per 1,000** (from 97 fewer to 218 more)
**First pass isolation**											
300 (2 RCTs)	serious^a^	not serious	not serious	very serious^d^	none	⨁◯◯◯ Very low	128/152 (84.2%)	122/148 (82.4%)	**RR 0.98** (0.88 to 1.08)	842 per 1,000	**17 fewer per 1,000** (from 101 fewer to 67 more)
**Total procedure time (min)**											
694 (6 RCTs)	serious^a^	very serious^e^	not serious	serious^f^	none	⨁◯◯◯ Very low	283	411	-	The mean total procedure time (min) was **0**	MD **22.88 lower** (36.13 lower to 9.63 lower)
**PVI time (min)**											
276 (3 RCTs)	serious^a^	not serious	not serious	not serious	none	⨁⨁⨁◯ Moderate	99	177	-	The mean PVI time (min) was **0**	MD **19.73 lower** (23.93 lower to 15.53 lower)
**Fluoroscopy time (min)**											
366 (3 RCTs)	serious^a^	very serious^e^	not serious	serious^f^	none	⨁◯◯◯ Very low	118	248	-	The mean fluoroscopy time (min) was **0**	MD **0.69 lower** (2 lower to 0.62 higher)
**Radiofrequency time (min)**											
484 (4 RCTs)	serious^a^	very serious^e^	not serious	not serious	none	⨁◯◯◯ Very low	202	282	-	The mean radiofrequency time (min) was **0**	MD **10.53 lower** (12.87 lower to 8.19 lower)
**Maximum temperature (°C)**											
276 (2 RCTs)	serious^a^	very serious^e^	not serious	very serious^f^	none	⨁◯◯◯ Very low	138	138	-	The mean maximum temperature (°C) was **0**	MD **3.91 higher** (0.98 higher to 6.84 higher)
**Safety—Any complications**											
691 (6 RCTs)	serious^a^	not serious	not serious	very serious^b^	none	⨁◯◯◯ Very low	8/283 (2.8%)	20/408 (4.9%)	**RR 1.15** (0.50 to 2.67)	28 per 1,000	**4 more per 1,000** (from 14 fewer to 47 more)
**Safety—Oesophageal lesion**											
541 (5 RCTs)	serious^a^	not serious	not serious	very serious^b^	none	⨁◯◯◯ Very low	6/233 (2.6%)	17/308 (5.5%)	**RR 1.15** (0.43 to 3.07)	26 per 1,000	**4 more per 1,000** (from 15 fewer to 53 more)

*CI* confidence interval; *MD* mean difference; *RR* risk ratio

Explanations

a. All of the included trials had overall some concerns of due to deviation from intended interventions, and Francke et al. with a high risk of selection bias

b. A wide confidence interval that does not exclude the appreciable benefit/harm, with a low number of events

c. I-square test > 50%

d. Low number of events

e. I-square test > 75%

f. A wide confidence interval that does not exclude the appreciable benefit/harm

### Primary outcomes

**Fig. 3:**
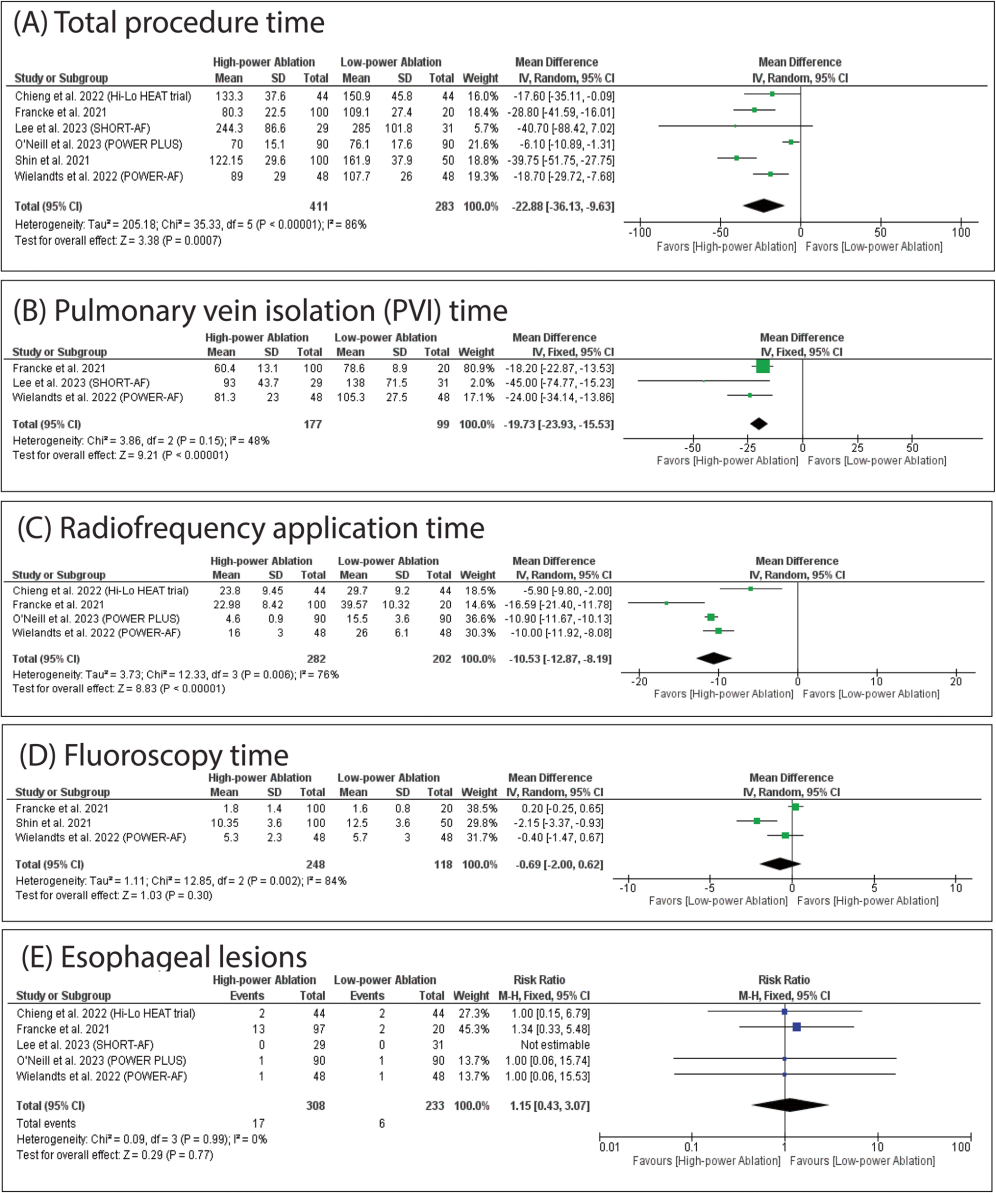
Forest plots of the primary outcomes (total procedure time, PVI time, radiofrequency (RF) application time, fluoroscopy time, and esophageal lesions), MD: mean difference, RR: risk ratio, CI: confidence interval

HPSD ablation was significantly associated with decreased total procedure time (MD: -22.88 with 95% CI [-36.13, -9.63], P = 0.0007) (Fig. 3A), PVI time (MD: -19.73 with 95% CI [-23.93, -15.53], P < 0.00001) (Fig. 3B), and radiofrequency application time (MD: -10.53 with 95% CI [-12.87, -8.19], P < 0.00001) (Fig. 3C). However, there was no significant difference between HPSD and SPLD ablation in fluoroscopy time (MD: -0.69 with 95% CI [-2.00, 0.62], *P* = 0.30) (Fig. 3D) and the incidence of esophageal lesions (RR: 1.15 with 95% CI [0.43, 3.07], *P* = 0.77) (Fig. 3E).

The pooled studies were homogenous in PVI time (I^2^ = 48%, *P* = 0.15) and esophageal lesions (I^2^ = 0%, *P* = 0.99). However, pooled studies were heterogeneous in total procedure time (I^2^ = 86%, *P* < 0.00001), radiofrequency application time (I^2^ = 76%, *P* = 0.006), and fluoroscopy time (I^2^ = 84%, *P* = 0.002). Regarding total procedure time and radiofrequency application time, heterogeneity was not resolved by leave-one-out sensitivity analysis. Regarding fluoroscopy time, heterogeneity was best resolved by excluding Shin et al. 2021 (I^2^ = 3%, *P* = 0.31) (Table S5).

Test for subgroup analysis based on the power used in the HPSD group was not significant across all outcomes (*P* > 0.1) (Figures S1-S5).

### Secondary outcomes

**Fig. 4:**
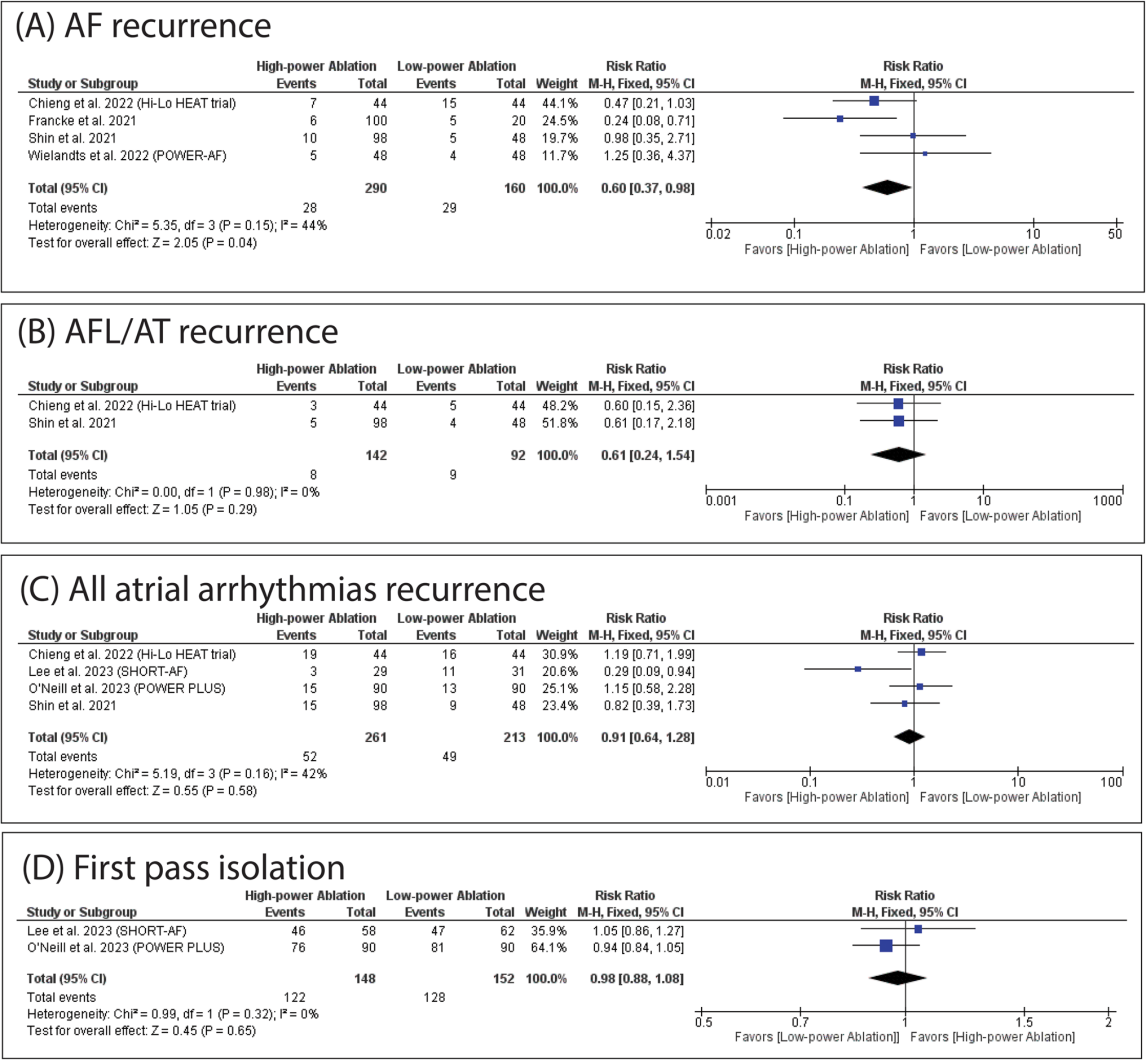
Forest plots of the secondary outcomes (AF recurrence, AFL/AT recurrence, all atrial arrhythmias recurrence, and first pass isolation), RR: risk ratio, CI: confidence interval

**Fig. 5:**
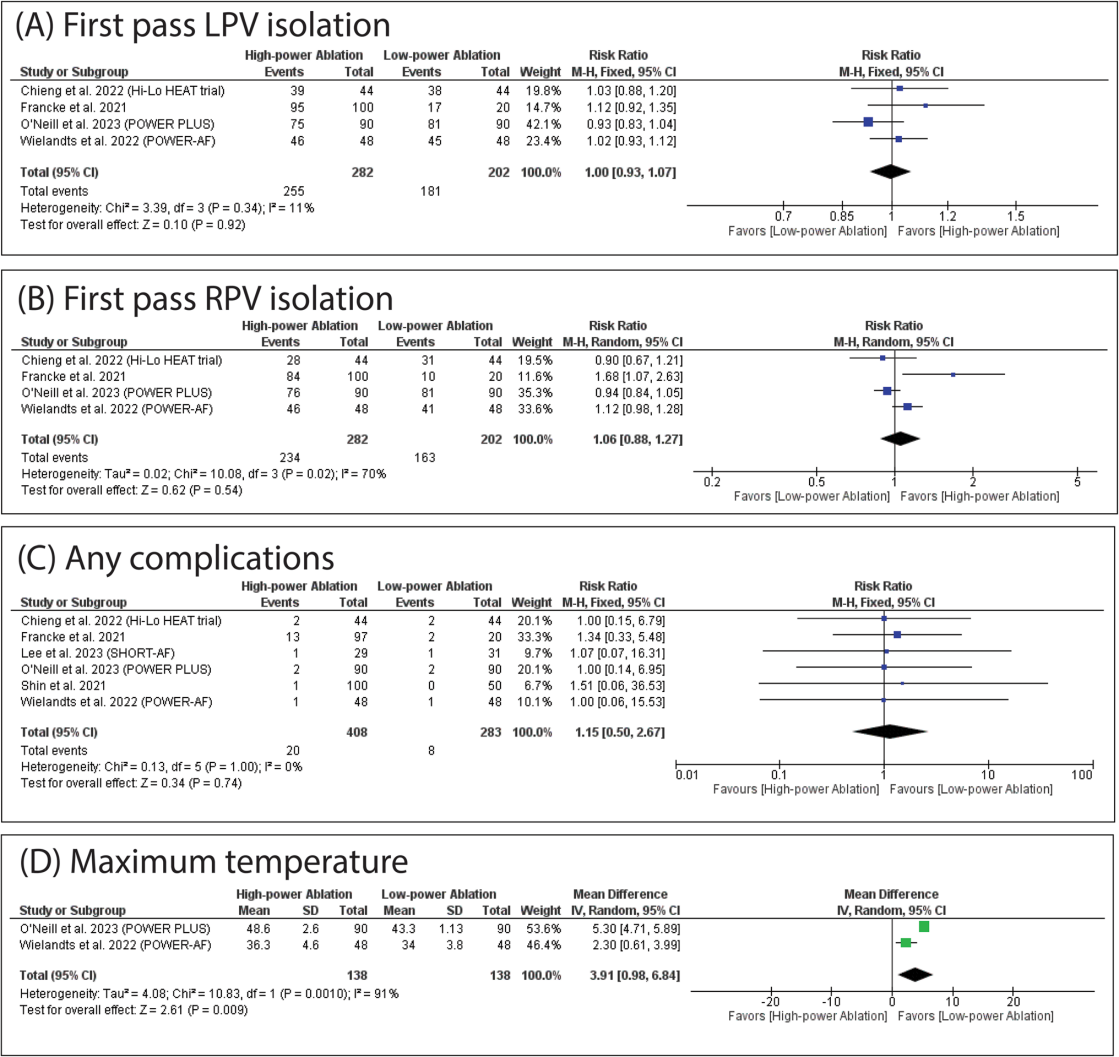
Forest plots of the secondary outcomes (first pass LPV isolation, first pass RPV isolation, any complications, and maximum temperature), RR: risk ratio, CI: confidence interval

HPSD was significantly associated with decreased incidence of AF recurrence (RR: 0.60 with 95% CI [0.37, 0.98], *P* = 0.04) (Fig. 4A). However, there was no significant difference between HPSD and SPLD ablation in the incidence of AFL/AT recurrence (RR: 0.61 with 95% CI [0.24, 1.54], *P* = 0.29) (Fig. 4B), the incidence of atrial arrhythmias recurrence (RR: 0.91 with 95% CI [0.64, 1.28], *P* = 0.58) (Fig. 4C), the incidence of first pass isolation (RR: 0.98 with 95% CI [0.88, 1.08], *P* = 0.65) (Fig. 4D), the incidence of first pass LPV isolation (RR: 1.00 with 95% CI [0.93, 1.07], *P* = 0.92) (Fig. 5A), the incidence of first pass RPV isolation (RR: 1.06 with 95% CI [0.88, 1.27], P = 0.54) (Fig. 5B), and the incidence of any complications (RR: 1.15 with 95% CI [0.50, 2.67], *P* = 0.74) (Fig. 5C). On the contrary, SPLD ablation was significantly associated with low maximum temperature (MD: 3.91 with 95% CI [0.98, 6.84], *P* = 0.009) (Fig. 5D).

The pooled studies were homogenous in AF recurrence (I^2^ = 44%, *P* = 0.15), AFL/AT recurrence (I^2^ = 0%, *P* = 0.98), atrial arrhythmias recurrence (I^2^ = 42%, *P* = 0.16), first pass isolation (I^2^ = 0%, *P* = 0.32), first pass LPV isolation (I^2^ = 11%, *P* = 0.34), and the incidence of any complications (I^2^ = 0%, *P* = 1.00). However, pooled studies were heterogeneous in first-pass RPV isolation (I^2^ = 70%, *P* = 0.02). Regarding first-pass RPV isolation, leave-one-out sensitivity analysis did not resolve heterogeneity (Table S5).

Test for subgroup analysis based on the power used in the HPSD group was not significant across all outcomes (*P* > 0.1) (Figures S6-S11**)**.

## Discussion

The important findings of our current investigation are 1) HPSD was significantly associated with reduced procedure time, PVI time, and RF application time; 2) No significant difference was observed between HPSD and SPLD ablation regarding fluoroscopy time; 3) No significant difference was found between HPSD and SPLD ablation with respect to esophageal lesions; 4) HPSD was significantly associated with a decreased incidence of AF recurrence compared to SPLD ablation; 5) No significant difference was observed between HPSD and SPLD ablation regarding the incidence of first-pass isolation or any complications.

Successful AF ablations aim to achieve electrical isolation of pulmonary veins by creating a transmural scar with minimal collateral tissue damage. Lesion quality is crucial for a durable PVI. The RF power, duration, contact force, and catheter stability determine the lesion characteristics, including its diameter and depth. Ablation index (AI) is a weighted formula incorporating power, duration, and contact force, which has been introduced to predict and quantify lesion quality, with RF power having the largest contributor to it [28–30].

HPSD ablation improves lesion quality by maximizing resistive heating and minimizing conductive heating. Additionally, collateral tissue injury with respect to the esophagus can primarily be reduced by minimizing conductive heating as well [11, 31]. Resistive heating is a direct form of energy that occurs immediately upon catheter-myocardium interaction and ceases with RF application termination. On the other hand, conductive heating is an indirect form of energy transfer that affects distant tissues and continues even after RF application for a few seconds [15].

In our analysis, despite the significant association between HPSD and reduced AF recurrence, no significant association was observed between HPSD and AFL/AT recurrence. This discrepancy might be attributed to the role of pulmonary veins as an essential source of AF, unlike AFL/AT. Consequently, optimizing PVI lesion quality would be beneficial in AF rather than AFL/AT.

There was some noted variability in the definitions of SPLD ablation and HPSD ablation across the included studies. While HPSD was most frequently defined as 40-50W power, O’Neill et al. utilized the QDOT MICRO catheter, specifically designed for HPSD ablation, delivering a notably high power (90w) over 4 s in a temperature-controlled mode), which did not significantly correlate with reduced arrhythmias recurrence [19].

Atrioesophageal fistula (AEF) is a feared complication of AF ablation with a mortality of 60–70%. The incidence of AEF is 0.1 to 0.25% among AF ablation procedures, and it represents the second most common cause of death following AF ablation procedures along with stroke [32–34]. Even though our results demonstrated a significant association between HPSD ablation and a higher maximum temperature, there was no significant difference between HPSD and SPLD ablation in the incidence of esophageal lesions, suggesting that the higher temperature with HPSD did not result in clinically significant esophageal lesions.

Safety of HPSD ablation was demonstrated by Winkle et al., who reported very low complication rates in 10,284 patients [13]. Additionally, Vassalo et al. reported similar safety, similar efficacy, and reduced procedural and RF time in their observational study comparing HPSD to SPLD ablation [35]. Dhillon et al. analysis, including 100 patients, demonstrated shorter procedure times, reduced PV reconnection, and similar recurrence compared to SPLD [36].

Esophageal injury is a major concern, especially during posterior wall ablation. A prospective study by Chen et al. reported esophageal lesions in 3.5% of 122 patients undergoing HPSD AF ablation [37]. Another prospective study by Muller et al. reported esophageal lesions in 6% of 953 patients undergoing HPSD AF ablations [38]. A non-randomized comparison by Kaneshiro showed no difference in the incidence of esophageal lesions among 271 patients (7% versus 8%). The mechanism behind the safety profile of HPSD AF ablation is thought to involve maximizing resistive heating and minimizing conductive heating [11, 31]. Using Kansas City Classification, Francke et al. reported esophageal lesions graded as two deep ulcers (Type 2B) in the standard group and 13 cases in the HPSD group, which were three erythema (Type 1), nine superficial ulcers (Type 2A) and one deep ulcer (Type 2B) [17], Wielandts et al. reported a superficial ulcer (Type 2A) in the control group and perforation without communication with the atria (Type 3A), and Chieng et al. reported all ETI cases as superficial ulcers (Type 2A) [21]. However, O’Neill et al. reported one esophageal ulcer in the SPLD group and one small superficial esophageal erosion in the HPSD group [19].

In addition, we found that four RCTs reported no incidence of stroke in both groups [18–21]. Moreover, Francke et al. and Wielandts et al. reported no incidence of steam pops [17, 21]. However, O’Neill et al. reported the incidence of steam pops in one case in the HPSD arm [19].

In the POWER-AF trial, a narrower safety margin for HPSD on the posterior wall was observed, suggesting the need for increased preventive measures during posterior wall ablation and thorough post-procedural follow-up, including endoscopic evaluation.

Moreover, a recent meta-analysis of 15 retrospective observational studies with a total of 2,718 patients found that HPSD was associated with higher freedom from atrial arrhythmias (OR 1.44, P = 0.009), shorter total procedure duration (mean difference -37.35 min, P < 0.001), decreased fluoroscopy duration (mean difference -5.23 min, P < 0.001), and reduced RFA time (mean difference -16.26 min, p < 0.001), with a similar safety profile compared to SPLD [39].

These findings align with our study, indicating that HPSD ablation has a superior efficacy in preventing AF recurrence with shorter procedure and RFA time. The reduction in procedure time contributes to lower anaesthesia time and decreased anaesthesia-related complications. Furthermore, minimizing instrumentation time in the left atrium lowers the risk of periprocedural stroke, which is the second most common cause of death after AF ablation along with AEF [34].

## Limitations

Our results must be interpreted cautiously, considering the Cochrane ROB2 tool. One of the six RCTs [17] was judged to have high concerns about bias arising from the randomization process. Additionally, five of the six RCTs [16, 18–21] were judged to have some concerns for bias arising from deviations in intended interventions. In addition, one of the six RCTs [18] was judged to have some concerns about bias arising from the randomization process. Lastly, two of the six RCTs [17, 21] were also judged to have some concerns about bias in selecting reported results.

Our study is limited by variations in SPLD ablation and HPSD ablation definitions across included RCTs. Specifically, the POWER PLUS trial compared very HPSD ablation at 90 W to hybrid ablation at 35–50 W, whereas other RCTs in our analysis employed 40–50 W in the interventional group, comparing it to standard ablation with 20–40 W. The POWER PLUS trial was the only study using the QDot catheter, contributing to the heterogeneity of this trial compared to all other included trials.

Most studies utilized an electroanatomic three-dimensional mapping system, with CARTO being the most commonly employed system. Generalizability of results to ablation procedures using alternative systems or without mapping may be limited.

There was some heterogeneity in the use of continuous intraprocedural esophageal temperature monitoring. While most studies employed temperature monitoring, Shin et al. and Francke et al. did not utilize any. Moreover, none of the studies reported differences in esophageal temperature spikes or alert rates between SPLD and HPSD ablation.

Subgroup analysis based on AF-type, paroxysmal versus persistent AF, was not applicable due to a lack of separate data for each AF-type.

## Implications for future research

Future research is required to investigate the optimal power settings for AF ablation, given the variation in power thresholds across studies. Additionally, working towards standardizing protocols for HPSD and SPLD ablation procedures is essential to facilitate comparison across studies. Future research should investigate patient-reported outcomes to assess the quality of life and symptom improvement following ablation procedures.

## Conclusion

Our systematic review and meta-analysis suggest that HPSD ablation is significantly associated with a decreased incidence of AF recurrence compared to SPLD ablation, with a comparable safety profile. HPSD ablation also significantly reduces procedure, PVI, and RF application time, with no significant difference in fluoroscopy time or the incidence of first-pass isolation. HPSD ablation could represent a safe and effective alternative to conventional SPLD ablation. On the contrary, SPLD ablation was significantly associated with low maximum temperature.

## Supplementary Information

Below is the link to the electronic supplementary material.

Supplementary file1 (DOCX 284 KB)

## Data Availability

Not applicable.
