# Supplementary material for: Efficacy and safety of high-power short-duration ablation for atrial fibrillation: a systematic review and meta-analysis of randomized controlled trials
Source: J Interv Card Electrophysiol. 2024 Mar 9;67(6):1445–61. doi: 10.1007/s10840-024-01782-2 (PMC11379757; doi:10.1007/s10840-024-01782-2)
Supplement: Supplementary file 1 — Supplementary file1 (DOCX 284 KB) [file 10840_2024_1782_MOESM1_ESM.docx]

**Supplementary material:**

**Title.**

**Efficacy and Safety of High-Power Short-Duration for Atrial Fibrillation: A Systematic Review and Meta-analysis of Randomized Controlled Trials.**

**Running Title.**

High-Power Short-Duration for Atrial Fibrillation.

**Authors.**

Ahmed Mazen Amin^1^, Ramy Ghaly^2^, Ahmed A. Ibrahim^3^, Mohamed Ahmed Ali^4^, Omar Almaadawy^5^, Amr Elzahaby^6^, Mohamed Abuelazm^6^, Basel Abdelazeem^7^, Muhammad Bilal Munir^8^.

**Affiliations.**

1. Faculty of Medicine, Mansoura University, Mansoura, Egypt.
2. Internal Medicine, University of Missouri-Kansas City, Kansas City, MO, USA
3. Faculty of Medicine, Menoufia University, Menoufia, Egypt.
4. Qena Faculty of Medicine, South Valley University, Qena, Egypt
5. Internal Medicine, MedStar Health, Baltimore, USA
6. Faculty of Medicine, Tanta University, Tanta, Egypt.
7. Department of Cardiology, West Virginia University, West Virginia, USA.
8. Section of Electrophysiology, Division of Cardiology, Department of Medicine, University of California Davis, Sacramento, CA, USA.

**Keywords.**

Atrial fibrillation; Radiofrequency; Pulmonary vein isolation; HPSD; LPLD.

**Contents:**

**Figures.**Figure S1: Total procedure time subgroup analysis based on the power used in HPSD arm.

Figure S2: Pulmonary vein isolation (PVI) time subgroup analysis based on the power used in HPSD arm.

Figure S3: Radiofrequency (RF) application time subgroup analysis based on the power used in HPSD arm.

Figure S4: Fluoroscopy time subgroup analysis based on the power used in HPSD arm.

Figure S5: Esophageal lesions subgroup analysis based on the power used in HPSD arm.

Figure S6: AF recurrence subgroup analysis based on the power used in HPSD arm.

Figure S7: atrial flutter (AFL)/ atrial tachycardia (AT) recurrence subgroup analysis based on the power used in HPSD arm.

Figure S8: Atrial arrhythmias recurrence subgroup analysis based on the power used in HPSD arm.

Figure S9: First pass left pulmonary vein (LPV) isolation subgroup analysis based on the power used in HPSD arm.

Figure S10: First pass right pulmonary vein (RPV) isolation subgroup analysis based on the power used in HPSD arm.

Figure S11: Any complications subgroup analysis based on the power used in HPSD arm.

**Tables.**Table S1: Search strategy.

Table S2: Summary characteristics (inclusion criteria, ablation guidance, and ablation target).

Table S3: Baseline characteristics (participants comorbidities).

Table S4: Description of risk of bias (ROB) assessment.

Table S5: Sensitivity analysis.


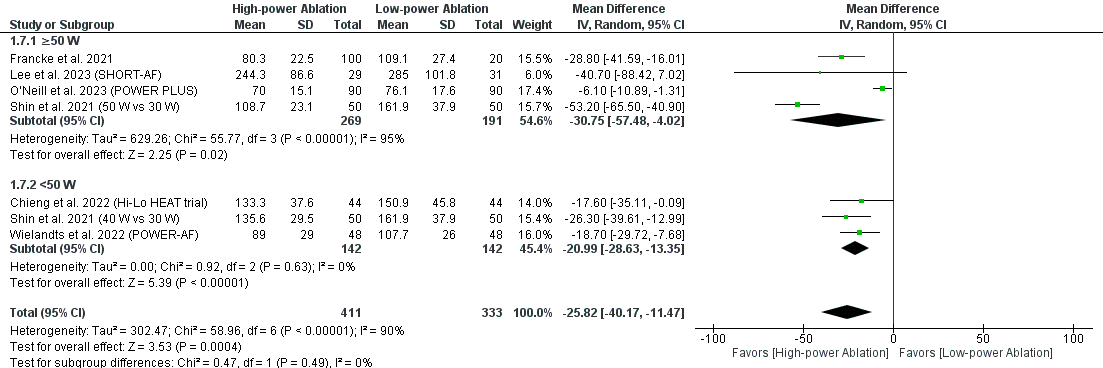


**Figure S1: Total procedure time subgroup analysis based on the power used in HPSD arm.**


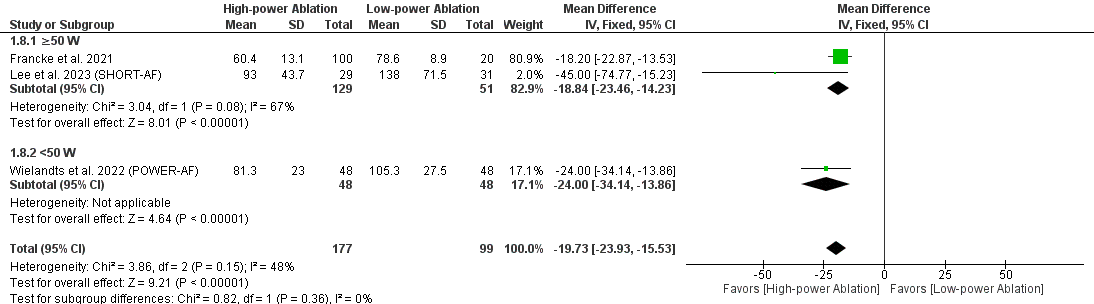


**Figure S2: Pulmonary vein isolation (PVI) time subgroup analysis based on the power used in HPSD arm.**


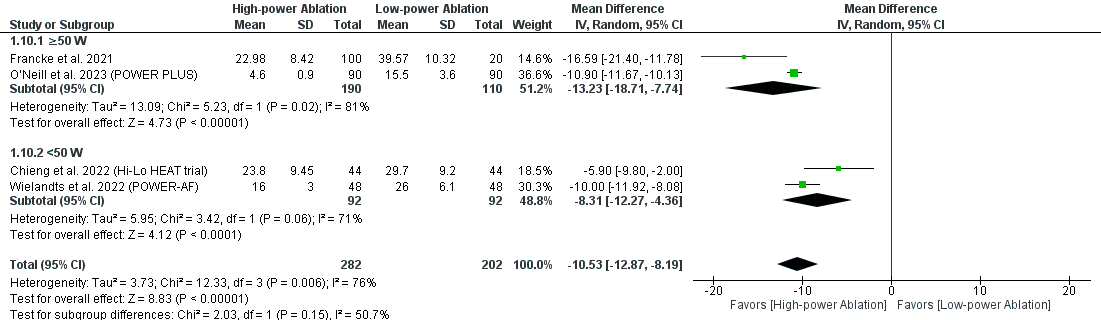


**Figure S3: Radiofrequency (RF) application time subgroup analysis based on the power used in HPSD arm.**


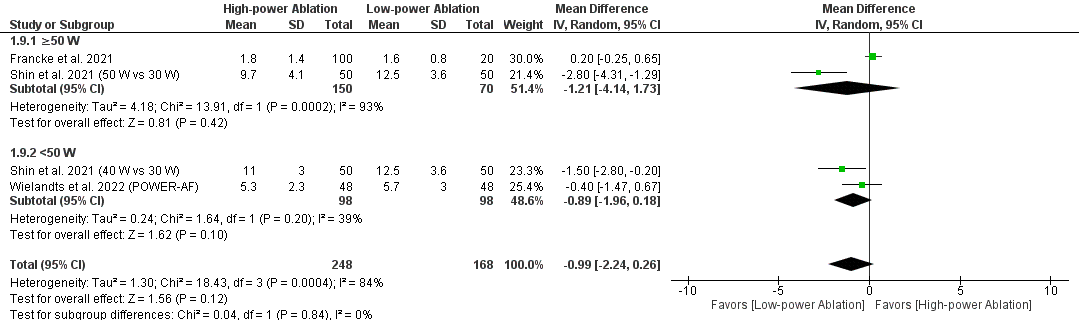


**Figure S4: Fluoroscopy time subgroup analysis based on the power used in HPSD arm.**


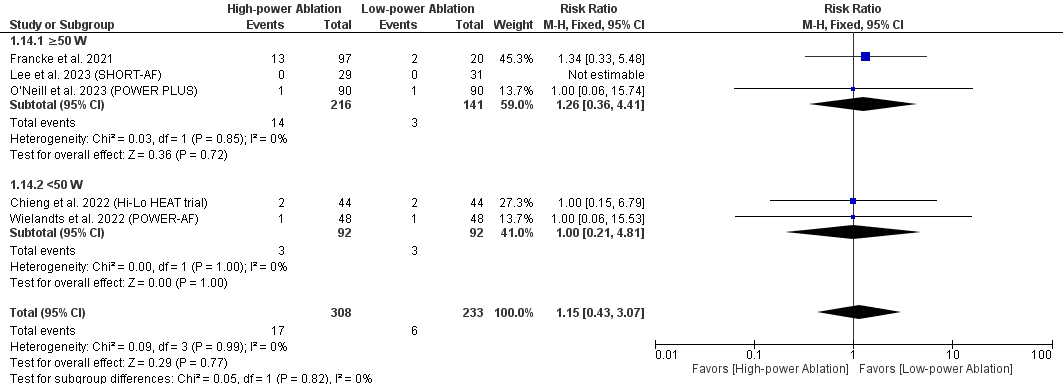


**Figure S5: Esophageal lesions subgroup analysis based on the power used in HPSD arm.**


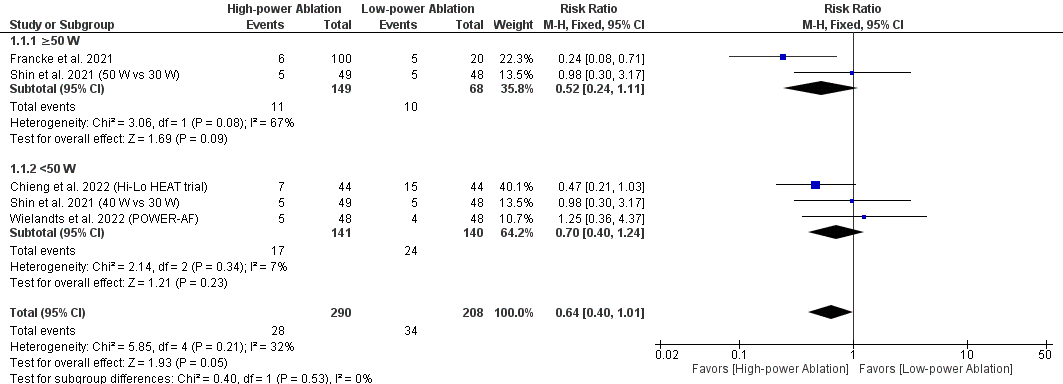


**Figure S6: AF recurrence subgroup analysis based on the power used in HPSD arm.**


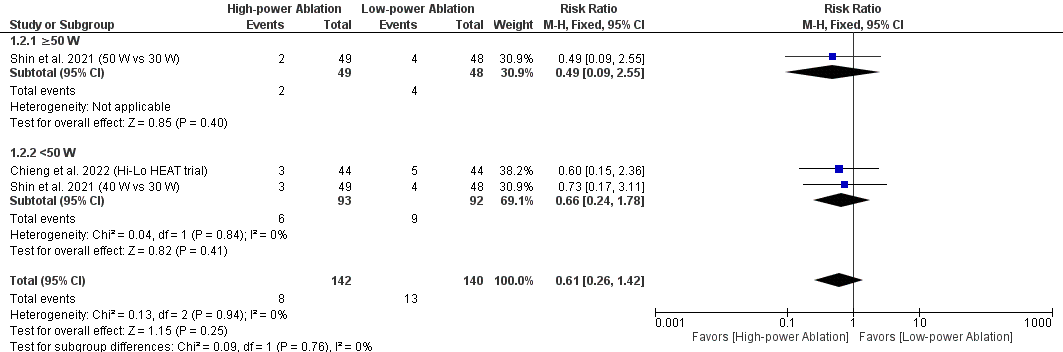


**Figure S7: atrial flutter (AFL)/ atrial tachycardia (AT) recurrence subgroup analysis based on the power used in HPSD arm.**


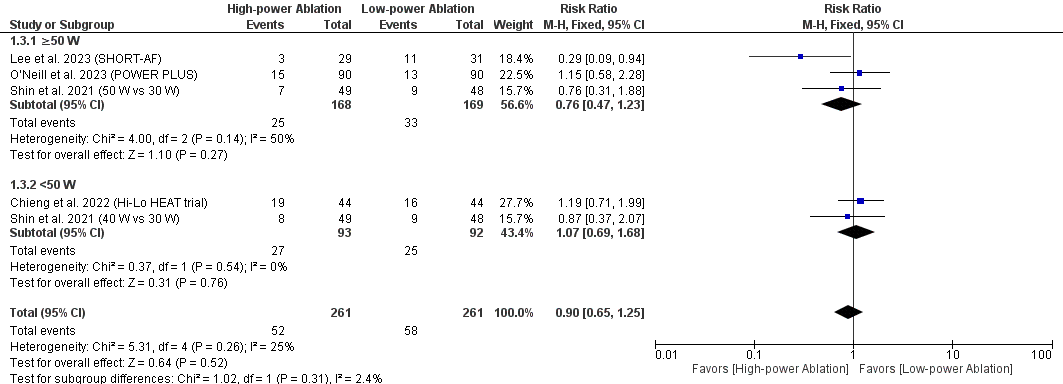


**Figure S8: Atrial arrhythmias recurrence subgroup analysis based on the power used in HPSD arm.**


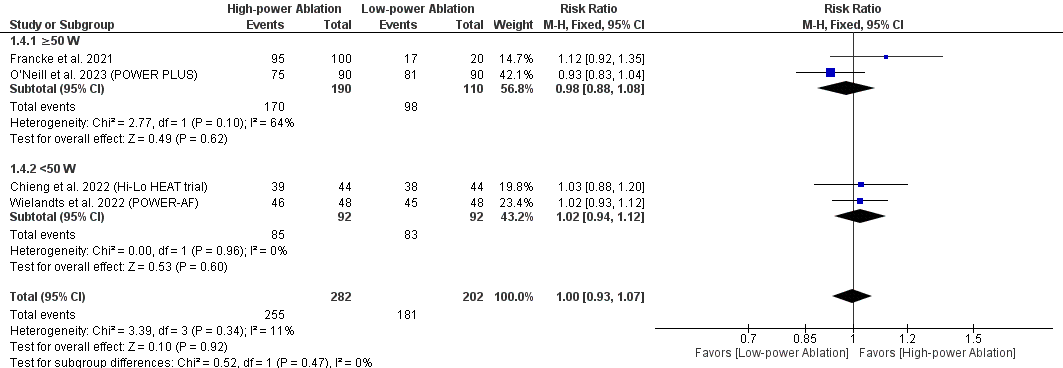


**Figure S9: First pass left pulmonary vein (LPV) isolation subgroup analysis based on the power used in HPSD arm.**


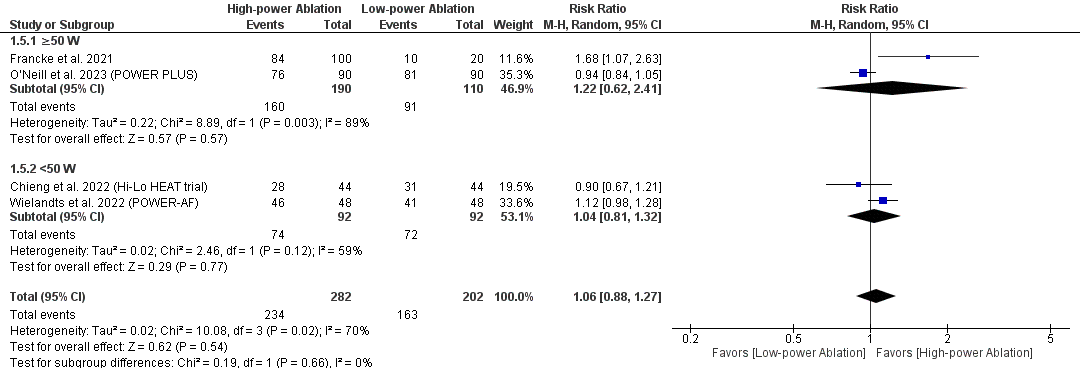


**Figure S10: First pass right pulmonary vein (RPV) isolation subgroup analysis based on the power used in HPSD arm.**


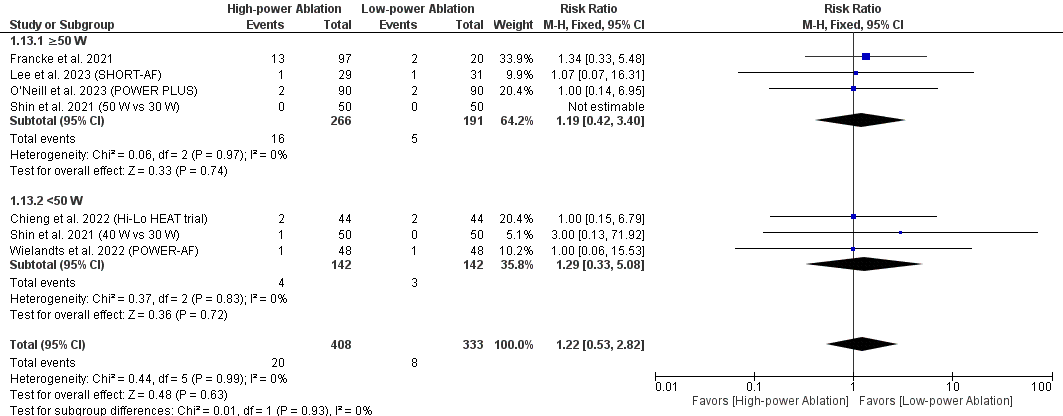


**Figure S11: Any complications subgroup analysis based on the power used in HPSD arm.**

| Database | Search Terms | Search Field | Search Results |
| --- | --- | --- | --- |
| Pubmed | (("High power*" OR "High-power*" OR "High-power ablation" OR "High-power shorter duration" OR HPSD) AND ("atrial fibrillation" OR AFib OR AF)) | All Field | 299 |
| Cochrane | (("High power*" OR "High-power*" OR "High-power ablation" OR "High-power shorter duration" OR HPSD) AND ("atrial fibrillation" OR AFib OR AF)) | All Field | 62 |
| WOS | (("High power*" OR "High-power*" OR "High-power ablation" OR "High-power shorter duration" OR HPSD) AND ("atrial fibrillation" OR AFib)) | All Field | 296 |
| SCOPUS | TITLE-ABS-KEY ( ( ( "High power*" OR "High-power*" OR "High-power ablation" OR "High-power shorter duration" OR hpsd ) AND ( "atrial fibrillation" OR afib OR af ) ) ) | Title, Abstract, Keywords | 408 |
| EMBASE | #3.  #1 AND #2                                                  469  #2.  'atrial fibrillation':ti,ab,kw OR afib:ti,ab,kw        200,289       OR af:ti,ab,kw  #1.  'high power*':ti,ab,kw OR 'high-power                   22,857       ablation':ti,ab,kw OR 'high-power shorter       duration':ti,ab,kw OR hpsd:ti,ab,kw | All Field | 469 |

**Table S1: Search Strategy.**

| **Study ID** | **Guidance** | **Ablation target** | **Main Inclusion Criteria** |  |
| --- | --- | --- | --- | --- |
|  |  |  |  |  |
| **Chieng et al. 2022 (Hi-Lo HEAT trial)** | Ablation index (AI) (CARTO, Biosense Webster), or lesion size index (LSI) (Ensite, Abbott Medical). | Anterior wall of the LA at 40–50 W power. Ablation targets were set at AI of 500–550, or LSI of 5–5.5. Left PV anterior antral line, ablation targets were set at AI of 484.5+/-28.7, or LSI of 5.1+/-0.2. Right PV anterior antral line, ablation targets were set at AI of 500.3+/-18.5, or LSI of 5.3+/-0.2. Left PV posterior antral line, ablation targets were set at AI of 382.4+/-20.2, or LSI of 4.2+/-0.4. Right PV posterior antral line, ablation targets were set at AI of 379.1+/-27.3, or LSI of 4.1+/-0.2. | Patients with paroxysmal AF (AF lasting <7 days) and persistent AF (AF lasting ≥7 days) undergoing their first catheter RF ablation procedure were recruited. |  |
| **Francke et al. 2021** | All procedures were guided by the Biosense Webster, Inc. Carto3 v7 mapping system with CARTOUNIVU® fluoroscopy integration. Ablation lesions were guided by the VISITAG SURPOINT® Module, with procedural guidelines and tag settings previously described and validated in the CLOSE protocol. | AI targets of 400 and 550 were used for posterior and anterior lesions, respectively, with an interlesion distance of less than 6 mm and VisiTag size of 3 mm. | Consecutive males or females above 18 years of age suffering from symptomatic drug‐resistant AF requiring catheter ablation. |  |
| **Lee et al. 2023 (SHORT-AF)** | Either the CARTO V7 (Biosense Webster) or EnSite Precision (Abbott) mapping systems. | For CARTO, a Surpoint ablation index of 450 to 550 on the anterior, LA appendage ridge, or septal aspects and 350 to 400 on the posterior aspects of the PV antra was targeted. For Ensite, a lesion size index of 5.5 to 6.0 on the anterior, LA appendage ridge, or septal aspect and 4.5 to 5.0 on the posterior aspects of the PV antra were targeted. | Eligible patients were at least 18 years of age and scheduled to undergo de novo PVI for either paroxysmal or persistent (<1year duration) AF |  |
| **O’Neill et al. 2023 (POWER PLUS)** | The CARTO-3, 3-dimensional mapping system and nMARQ RF generator (Biosense Webster, Diamond Bar, California). | AI values were targeted to >=550 at the anterior wall and >=400 posteriorly, and at the roof and inferior aspect of the veins with an inter-tag distance of #6 mm. | Patients with paroxysmal or persistent atrial fibrillation undergoing first-time PVI |  |
| **Shin et al. 2021** | We used the CARTOVR3 three-dimensional electroanatomical mapping system (Biosense Webster Inc., Irvine, CA, USA) and a fivesplined catheter (PENTARAYVR, Biosense Webster Inc., Irvine, CA, USA) to obtain the geometry and voltage mapping of the left atrium (LA) and PVs. An irrigated CF-sensing catheter (THERMOCOOL SMARTTOUCHVR SF, Biosense Webster Inc., Irvine, CA, USA) was used to obtain quantitative ablation lesion parameters. | Radiofrequency was delivered targeting inter-lesion distance of <_6mm in all patients. anterior, roof, and inferior segments of pulmonary vein (PV) antra and roof line between each upper PV | patients undergoing first-time RF catheter ablation (RFCA) for AF [paroxysmal AF (PAF) and non-PAF] |  |
| **Wielandts et al. 2022 (POWER-AF)** | CLOSE-guided pulmonary vein isolation using a contact force radiofrequency catheter | NA | paroxysmal AF, planned for first CLOSE-guided pulmonary vein isolation using a contact force radiofrequency catheter |  |

**Table S2: Summary characteristics (inclusion criteria, ablation guidance, and ablation target)**

ETI: esophageal thermal injury, HPSD: high-power short-duration, LPLD: low-power long-duration, RF: radio-frequency, PVI: pulmonary vein isolation, AI: ablation index, AF: atrial fibrillation, LA: left atrium, lesion size index (LSI), PV: pulmonary vein, NA.: not available.

| **Study ID** | **Comorbidities N.(%)** | | | | | | | | | |
| --- | --- | --- | --- | --- | --- | --- | --- | --- | --- | --- |
|  | **Hypertension** | | **Diabetes** | | **IHD or CAD** | | **Obstructive sleep apnea** | | **Stroke** | |
|  | **Intervention** | **control** | **Intervention** | **control** | **Intervention** | **control** | **Intervention** | **control** | **Intervention** | **control** |
| **Chieng et al. 2022 (Hi-Lo HEAT trial)** | 25 (56.8) | 14 (31.8) | 3 (6.8) | 2 (4.5) | 5 (11.4) | 7 (15.9) | 7 (15.9) | 2 (4.5) | 1 (2.3) | 2 (4.5) |
| **Francke et al. 2021** | NA | NA | NA | NA | NA | NA | NA | NA | NA | NA |
| **Lee et al. 2023 (SHORT-AF)** | 12 (41) | 19 (61) | 2 (7) | 4 (13) | 5 (17) | 7 (23) | 5 (17) | 9 (29) | 2 (7) | 3 (10) |
| **O’Neill et al. 2023 (POWER PLUS)** | 41 (45.6) | 38 (42.2) | 8 (8.9) | 7 (7.8) | 16 (17.8) | 12 (13.3) | NA | NA | NA | NA |
| **Shin et al. 2021** | 56 (56) | 22 (44) | 21 (21) | 8 (16) | NA | NA | NA | NA | 12 (12) | 6 (12) |
| **Wielandts et al. 2022 (POWER-AF)** | NA | NA | NA | NA | NA | NA | NA | NA | NA | NA |

**Table S3: Baseline characteristics (participants comorbidities).**

N: number, IHD: ischemic heart disease, CAD: coronary artery disease, NA: not available.

| **Study ID** | **Domain** | **Decision** | **Description** |
| --- | --- | --- | --- |
|  |  |  |  |
| **Chieng et al. 2022 (Hi-Lo HEAT trial)** | Randomization process | Low risk | Treatment group randomization was computer-generated. Double blinding was utilized, with participants and clinicians involved in participant follow-up blinded to treatment allocation, and there were no apparent differences between the two groups. |
|  | Deviations from intended interventions | Some concerns | Double blinding was utilized, with participants and clinicians involved in participant follow-up blinded to treatment allocation (Participants and assessors were blinded, while the operator was unblinded), but there is no information about the analysis method if it was intention to treat or other methods. |
|  | Missing outcome data | Low risk | Outcome data of nearly all randomized patients were available. |
|  | Measurement of the outcome | Low risk | Appropriate tools were used to measure the outcome without difference between the two group arms. |
|  | Selection of the reported result | Low risk | All outcomes, measurement tools, and analysis plans were pre-specified in the study protocol. |
|  | **OVERALL** | **SOME CONCERNS** | |
| **Francke et al. 2021** | Randomization process | High risk | Randomization with a blinded draw for only 40 patients out of 120.  Concealed allocation through sealed envelopes is done for only randomized patients, and there is a big difference between the two arms’ sample size. |
|  | Deviations from intended interventions | Some concerns | Only randomized patients were blinded. |
|  | Missing outcome data | Low risk | Outcome data were available for nearly all participants. |
|  | Measurement of the outcome | Low risk | Appropriate tools were used to measure the outcome without difference between the two group arms. |
|  | Selection of the reported result | Some concerns | No information about whether the outcomes and the analysis methods were pre-specified. |
|  | **OVERALL** | **HIGH RISK** | |
| **Lee et al. 2023 (SHORT-AF)** | Randomization process | Some concerns | There was no information about how they randomized the patients and if they were concealed. |
|  | Deviations from intended interventions | Some concerns | However, the participants were blinded, but there is no information about the analysis method if it was intention to treat or other methods. |
|  | Missing outcome data | Low risk | Outcome data were available for nearly all participants. |
|  | Measurement of the outcome | Low risk | Appropriate tools were used to measure the outcome without difference between the two group arms. |
|  | Selection of the reported result | Low risk | data that produced this result analysed in accordance with a pre-specified analysis plan. |
|  | **OVERALL** | **SOME CONCERNS** | |
| **O’Neill et al. 2023 (POWER PLUS)** | Randomization process | Low risk | Patients were randomized in a 1:1 manner (a priori and concealed until the time of the procedure) |
|  | Deviations from intended interventions | Some concerns | The participants were aware of their assigned group because it is an unblinded study, and they used the per-protocol analysis method. |
|  | Missing outcome data | Low risk | Outcome data were available for nearly all participants. |
|  | Measurement of the outcome | Low risk | Appropriate tools were used to measure the outcome without difference between the two group arms. |
|  | Selection of the reported result | Low risk | data that produced this result analysed in accordance with a pre-specified analysis plan. |
|  | **OVERALL** | **SOME CONCERNS** | |
| **Shin et al. 2021** | Randomization process | Low risk | Central randomization at a 1:1:1 ratio was performed using computer-generated random permutation sequences. |
|  | Deviations from intended interventions | Some concerns | The participants were blinded, but there is no information about the analysis method if it was intention to treat or other methods. |
|  | Missing outcome data | Low risk | Outcome data were available for nearly all participants. |
|  | Measurement of the outcome | Low risk | Appropriate tools were used to measure the outcome without difference between the two group arms. |
|  | Selection of the reported result | Low risk | data that produced this result analysed in accordance with a pre-specified analysis plan. |
|  | **OVERALL** | **SOME CONCERNS** | |
| **Wielandts et al. 2022 (POWER-AF)** | Randomization process | Low risk | Overall, 100 patients (88+12, 15% margin for lost data) were pre-randomized using a 1:1 block randomization technique with patient allocation in concealment done at enrollment. |
|  | Deviations from intended interventions | Some concerns | There is no information about blinding status and there is no information about the analysis method if it was intention to treat or other methods. |
|  | Missing outcome data | Low risk | Outcome data were available for nearly all participants. |
|  | Measurement of the outcome | Low risk | Appropriate tools were used to measure the outcome without difference between the two group arms. |
|  | Selection of the reported result | Some concerns | data that produced this result analysed in accordance with a pre-specified analysis plan. |
|  | **OVERALL** | **SOME CONCERNS** | |

**Table S4: Description of risk of bias (ROB) assessment.**

| Outcome | No. of  Participants (/) | No. of  trials | Quantitative data synthesis | | | | Heterogeneity analysis | | |
| --- | --- | --- | --- | --- | --- | --- | --- | --- | --- |
|  |  |  | MD | 95% CI | Z value | p-value | df | p-value | I2 (%) |
| **First pass RPV (%).** | | | | | | | | | |
| Chieng et al. 2022 (Hi-Lo HEAT trial) | 238/158 | 3 | 1.11 | [0.89, 1.40] | 0.92 | 0.36 | 2 | 0.006 | 80% |
| Francke et al. 2021 | 182/182 | 3 | 1.00 | [0.87, 1.16] | 0.01 | 0.99 | 2 | 0.08 | 60% |
| O’Neill et al. 2023 (POWER PLUS) | 192/112 | 3 | 1.14 | [0.88, 1.48] | 0.98 | 0.33 | 2 | 0.06 | 64% |
| Wielandts et al. 2022 (POWER-AF) | 234/154 | 3 | 1.06 | [0.78, 1.45] | 0.39 | 0.70 | 2 | 0.02 | 74% |
| **Total procedure time (min).** | | | | | | | | | |
| Chieng et al. 2022 (Hi-Lo HEAT trial) | 367/239 | 5 | -24.09 | [-39.52, -0.66] | 3.06 | 0.002 | 4 | 0.0001 | 89% |
| Francke et al. 2021 | 311/263 | 5 | -21.64 | [-36.78, -6.50] | 2.80 | 0.005 | 4 | 0.0001 | 86% |
| Lee et al. 2023 (SHORT-AF) | 382/252 | 5 | -21.81 | [-35.51, -8.11] | 3.12 | 0.002 | 4 | 0.0001 | 88% |
| O’Neill et al. 2023 (POWER PLUS) | 321/193 | 5 | -27.29 | [-36.98, -17.59] | 5.52 | 0.001 | 4 | 0.09 | 50% |
| Shin et al. 2021 | 311/233 | 5 | -18.01 | [-29.22, -6.80] | 3.15 | 0.002 | 4 | 0.004 | 74% |
| Wielandts et al. 2022 (POWER-AF) | 363/235 | 5 | -24.28 | [-41.31, -7.24] | 2.79 | 0.005 | 4 | 0.0001 | 88% |
| **Fluoroscopy time (min).** | | | | | | | | | |
| Francke et al. 2021 | 148/98 | 2 | -1.25 | [-2.96, 0.47] | 1.43 | 0.15 | 1 | 0.03 | 78% |
| **Shin et al. 2021** | 148/68 | 2 | 0.11 | [-0.32, 0.53] | 0.48 | 0.63 | 1 | 0.31 | 3% |
| Wielandts et al. 2022 (POWER-AF) | 200/70 | 2 | -0.90 | [-3.20, 1.40] | 0.77 | 0.44 | 1 | 0.0004 | 92% |
| **Radiofrequency time (min).** | | | | | | | | | |
| Chieng et al. 2022 (Hi-Lo HEAT trial) | 238/158 | 3 | -11.34 | [-13.39, -9.30] | 10.8 | <0.0001 | 2 | 0.04 | 68% |
| Francke et al. 2021 | 182/182 | 3 | -9.71 | [-11.72, -7.71] | 9.49 | <0.0001 | 2 | 0.04 | 69% |
| O’Neill et al. 2023 (POWER PLUS) | 192/112 | 3 | -10.59 | [-15.37, -5.81] | 4.34 | <0.0001 | 2 | 0.003 | 83% |
| Wielandts et al. 2022 (POWER-AF) | 234/154 | 3 | -10.91 | [-15.31, -6.52] | 4.87 | <0.0001 | 2 | 0.003 | 83% |

**Table S5: Sensitivity analysis**

MD: mean difference; CI: confidence interval; df: degrees of freedom.
